# A Comprehensive Account of the Rust Genus *Skierka* (*Skierkaceae*)

**DOI:** 10.3390/jof8121243

**Published:** 2022-11-24

**Authors:** Acharya Balkrishna, Ajay Kumar Gautam, Shubhi Avasthi, Mekala Niranjan, Rajnish Kumar Verma, Vedpriya Arya, Ashwani Kumar, Samantha C. Karunarathna, Nakarin Suwannarach

**Affiliations:** 1 Patanjali Herbal Research Department, Patanjali Research Institute, Haridwar 249405, India; 2 Department of Applied and Allied Sciences, University of Patanjali, Haridwar 249405, India; 3 School of Studies in Botany, Jiwaji University, Gwalior 474011, India; 4 Department of Botany, Rajiv Gandhi University, Itanagar 791112, Arunachal Pradesh, India; 5 Mycology Lab., Department of Plant Pathology, Punjab Agricultural University, Ludhiana 141004, India; 6 Center for Yunnan Plateau Biological Resources Protection and Utilization, College of Biological Resource and Food Engineering, Qujing Normal University, Qujing 655011, China; 7 Research Center of Microbial Diversity and Sustainable Utilization, Chiang Mai University, Chiang Mai 50200, Thailand

**Keywords:** diversity, distribution, molecular phylogeny, *Pucciniomycetes*, *Skierka* rust, taxonomy

## Abstract

The rust genus *Skierka* belonging to the phylum *Basidiomycota* was described in 1900 by Raciborski with *Skierka canarii* as the type species. The published literature on this rust genus reveals its ambiguity in taxonomic placement. It was challenging to taxonomically delineate and precisely identify each species within this genus due to the species sharing some common characteristics. The latest studies based on morphology taxonomy and molecular characteristics, however, have solved this puzzle now and placed this genus in its new family *Skierkaceae*. To understand all about the genus *Skierka,* this compilation was carried out to unveil the general characteristics, history, diversity, distribution, ecology, morphology and molecular taxonomy of different species of *Skierka*. After exploring 14 species of *Skierka*, it was observed that this genus is distributed in seven plant families in 19 countries all over the world. The genus appears to be well-represented in Asian and South American counties. This rust has not been reported from any European countries to date. The morpho-taxonomy of all species is well studied, but molecular analyses are still required. Only two species of the genus namely *S. robusta* and *S. diploglottidis* were identified based molecular analyses. Therefore, further studies should be focused on epitypifying the taxa that are too old and updating their taxonomy based on molecular, biochemical, and physiological aspects along with morphological characteristics. Multiple analytical methods should be considered when dealing with multi-locus datasets. This will increase our understanding of the diversity, distribution, and identification of these rust fungi.

## 1. Introduction

Fungi, one of the most diverse creatures on planet Earth, play a significant structural and functional role in many ecosystems. After insects, these creatures comprise the biggest group in a variety of habitats, such as soil, water, air, animals, plants, and ecosystems, with harsh conditions such as low or high temperature, and high concentrations of metals and salts. Fungi display a diverse spectrum of genus and species diversity due to their capacity to exist in a vast array of morphologies, lifestyles, and developmental patterns. Because of the crucial functions that fungi play in ecosystem function, it has become essential to investigate their diversity and distribution over the world [1]. Several researcher groups are exploring and characterizing the diversity of fungi, using a range of basic and advanced techniques. With an estimated 1.5 million fungal species on Earth [2], fungal species numbers are now estimated to range from 2.2 to 3.8 million depending on host association [3,4] and 11.7 to 13.2 million utilizing high-throughput sequencing. Despite the vast diversity of fungi, only around 150,000 fungal species have been reported to date [4].

Rust fungus makes up one of the largest groupings of fungi among all other groups. It constitutes one of the largest fungal orders *Pucciniales*, which is comprised of seven suborders, 18 families and more than 7000 species [5,6]. With a vast diversity of species, rust fungi form the most species-rich group of plant pathogens. The diseases caused by rust fungi are among the earliest recognized diseases of agricultural plants [7]. However, rusts are regarded as one of the fungal groups whose taxonomy always remains problematic. A number of attempts have been made to resolve the taxonomic ambiguity of rust fungi. A higher-rank classification for rust fungi was recently provided by Aime and McTaggart [6], with the proposal to resolve some existing taxonomic confusions by proposing some new taxonomic ranks. This higher-rank classification resolves the taxonomic placement of the rust genus *Skierka* in a new suborder *Skierkineae* and a new family *Skierkaceae*. The present study is compiled to provide complete information about the rust genus *Skierka*. 

The goal of the current publication is to provide readers with a current understanding of the rust genus *Skierka*, as well as extensive information on the taxonomic framework, history, diversity and distribution, ecology, and molecular diversity. Detailed descriptions of each species of the genus *Skierka* that have been recorded so far are given to cover it completely in terms of taxonomic updates. To investigate the intergeneric relationships of *Skierka*, phylogenetic analyses were also performed by using DNA sequence data of several gene regions that were accessible in GenBank and published literature.

## 2. The Genus *Skierka*

### 2.1. General History

The *Skierka* is a rust genus of the phylum *Basidiomycota*, family *Skierkaceae*, order *Pucciniales*, class *Pucciniomycetes* and subphylum *Pucciniomycotina*. This genus was described in 1900 by Raciborski [8] and typified as *Skierka canarii* Racib. The other species of *Skierka* were reported and described subsequently as *S. congonensis* from Africa [9], *S*. *agallochoa* from Java [10], *S*. *holwayi* from Central America [11], and *S. robusta* from Africa [12]. A rust infection on *Cupania belizensis* reported in 1936 was identified as the telial stage of *Skierka*. Earlier, this rust on *C. belizensis* was identified as the telial stage of *Ctenoderma cristata,* which was later identified as the uredial stage of *Skierka*. A total of 14 *Skierka* species have been discovered so far on different plant hosts in different time intervals (Table 1). 

The classification of rust fungi was earlier based on the characteristics of basidia and teliospores. About three to four rust families, namely *Melampsoraceae*, *Coleosporiaceae*, *Pucciniaceae*, and *Zaghouaniaceae,* were proposed initially [13,14]. However, the additions of subfamilies and/or tribes based on the morphology of telia were proposed by Arthur [15], Sydow and Sydow [13], and Dietel [16]. Later on, emphasis on the gametothallus, especially spermogonial morphology and then its combination with teliospore morphology [1,17,18] was laid down, which resulted in the most broadly applied 13 family rust classification. Based on such observations, the genus *Skierka* was initially placed in the family *Pucciniaceae* by most authors, despite the fact of it having characteristic sessile and adhering teliospores [8,13]. The uncertainty about the taxonomic position of this rust genus remained. Later on, Arthur [15] proposed a subfamily *Skierkatae* of the *Aecidiaceae* (*Pucciniaceae*) where three other genera, along with *Skierka*, were also included. However, considering that the teliospore characteristics formed the major basis of this placement, the relationship between *Skierka* and the other genera was not so encouraging as to place them together. Subsequently, Dietel [16] placed *Skierka* in the tribe *Skierkeae* of the *Pucciniaceae*, while *Hemileieae* also proposed its inclusion in this tribe based on developing their sori in relation to the stomata of their hosts. He also suggested the similarity between the genus *Spirechina* and *Uredinopsis* but was not able to fully justify supporting this similarity. The genus *Skierka* was also placed in the *Melampsoraceae* by Koorders [19], but the characteristic teliospores of this rust genus did not fully support it. In addition, characteristics, such as the absence of pedicels and lateral adherence of the teliospores of *Skierka* differentiated it from genera of *Melampsoraceae*. The similarity between the genus *Cronartium* and *Skierka* was also considered once, but a detailed investigation considered it a superficial similarity. It seems evident that *Skierka* represents a distinct line of development and should be placed in a tribe *Skierkeae* by itself. The species of *Skierka* are observed to be tropical and autoecious [1,20], producing sub-epidermal and deep-seated; non-catenulate teliospores that extruded in hair-like columns. Although other researchers placed *Skierka* in a separate subfamily or tribe, Mains [20] considered it an intermediate taxon between *Melampsoraceae* and *Pucciniaceae*. However, Cummins and Hiratsuka [1] treated it as *incertae sedis* within the rusts. 

With the inclusion of the combined characteristics, morphology of gametothallus, basidia, and teliospores, a 13 family classification was proposed, which became the most broadly applied in the pre-molecular era; here, the genus *Skierka* was placed under *Pileolariaceae* along with the genus *Pileolaria*. With the use of molecular systematic study, several taxonomic ambiguities at the level of family, genus or even species have been resolved to large extent [21]. Recently, a higher-rank classification for rust fungi has been proposed by Aime and McTaggart [6], based on 16 years of sampling that includes ca. 80% of accepted genera including type species and three gene loci of DNA, to resolve the deeper nodes of the rust fungus tree of life. In this high-rank taxonomic framework, the new suborder *Skierkineae* Aime and McTaggart, and the new family *Skierkaceae* (Arthur) Aime and McTaggart were proposed and placed the rust genus *Skierka* at its correct taxonomic position. A detailed event-wise history of this rust genus is presented here in Table 1. 

### 2.2. Diversity and Distribution 

**Figure 1:**
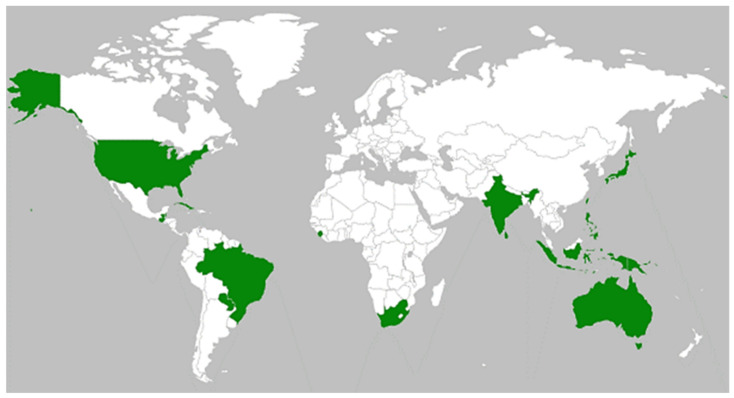
Map showing the global distribution of the described *Skierka* species.

The genus *Skierka* is predominantly present in the countries of Asian continents. The distribution of this genus has been reported in all continents except Europe. After the compiled information on diversity and distribution, it is found that the rust genus *Skierka* comprised a total of 14 species that occurred as obligate parasitic fungi on vascular plants in about 19 countries of the world. The percentage distribution of this rust genus was predominantly found in Asia (31.58%), which was followed by South America (21.15%), North America and Africa (15.79%, respectively), Oceania (10.53%), and Australia (5.27%). The genus appears to be well-represented in Asian and South American countries. In context to the number of records reported from different continents, the highest eight number was observed from Asian countries, followed by the countries of content North America, South America and Africa (four each), Oceania (three) and Australia with a single record. It was found to be distributed in a total of six countries in Asia, four in South America, three in North America and Africa, respectively, two in Oceania, and only one in Australia. A similar trend was observed for the occurrence of species in different countries and continents. When we analyzed the distribution of the species of *Skierka* in different plant families, it was observed in a total of seven families. The highest six species of *Skierka* were found reported in the plant family *Sapindaceae*, followed by *Burseraceae* (three species), *Euphorbiaceae* (two species), and a single species in the remaining four families. Similarly, when we compare the number of records of rust disease caused by species of *Skierka* on plant families, a maximum of eleven records were found on *Sapindaceae*, which was followed by *Burseraceae* (six), *Euphorbiaceae* (three); whereas, single records were found in the remaining host families. The array of this distribution of *Skierka* species reveals that this genus consists of 15 species distributed over 19 countries of the world, and this genus is still not so wide in distribution when compared to other major genera of rust fungi. The detailed list of described *Skierka* species, together with the host (family), and country (continent) of distribution is provided in Figure 1 and Table 2.

### 2.3. Ecology 

*Skierka* species are tropical and autoecious [1,20,35] and infect both shrubs and tree species. A total of seven plant families were observed to become infected by the species of this rust genus. A total of seven plant genera of the family *Sapindaceae* (*Cupania americana*, *C. belizonsis*, *C. macrophylla, Dictyoneura obtuse*, *Diploglottis* sp., *Matayba guianensis*, *Thouinidium decandrum*, *Litchi chinensis*, and *Sapindus bifoliolatus*) were found to be infected by the species of this rust genus, while four species of the genus *Canarium* in the family *Burseraceae* (*Canarium commune*, *C. moluccanum*, *C. luzonicum*, and *Canarium* sp.); three genera, namely *Excoecaria agallocha*, *Alchornea cordifolia* and *Macaranga* sp. in *Euphorbiaceae*; and only a singly genus in *Pistaceaceae* (*Pistacia integerrima*), *Rutaceae* (*Toddalia aculeate*), *Sterculiaceae* (*Dombeya* sp.), and *Vitaceae* (*Rhoicissus rhomboidea*). With respect to wide distribution, the Asian continent was predominant, with different species of *Skierka*. The reports of 15 species over 19 countries of the world justify their adaptation to different geographical regions and climatic conditions of the globe. However, no report from any European region revealed the non-preference of this rust in these areas. However, their discovery in future cannot be ignored. It is hard to conclude host generalism or specialism in *Skierka*, but for most *Skierka* species, multiple host trees are not suggested. In all species of *Skierka,* only *S. canarii* and *S. diploglottidis* showed an association with multiple plant genera while the rest of species were found to be confined to a single host plant genus. However, multiple species of many plant genera were found to be infected with a single species of rust fungi (Figure 1 and Table 2). The discussion about host specificity of the few species that seem to be connected with a single host will have to wait, as there are not many species recorded in this genus. 

## 3. Phylogeny and Molecular Diversity 

A phylogenetic study on 16 years of sampling of rust fungi based on three DNA loci was carried out to provide a taxonomic framework to resolve the deeper nodes of the rust fungus tree of life. As per this taxonomic framework, the order *Pucciniales* comprised seven suborders and 18 families. They newly defined higher ranks with consideration of the morphology, host range, and life cycle. Based on a phylogenetic analysis along with morphology, host range, and the life cycle a new family, *Skierkaceae* was introduced to accommodate the type genus *Skierka* in *Pucciniales*. As per the authors, deep-seated and subepidermal sporothalli sori with mature single-celled and non-catenulate uredinio and teliospores are forced through a narrow sorus opening by the production of new spores from sporogenous cells, from which they are detached before extrusion, differentiated this family from the others [6]. A total of 14 epithets are available on index fungorum, of which, the molecular data is only available for two species of *Skierka*, i.e., *S. diploglottidis* and *S. robusta*.

**Figure 2:**
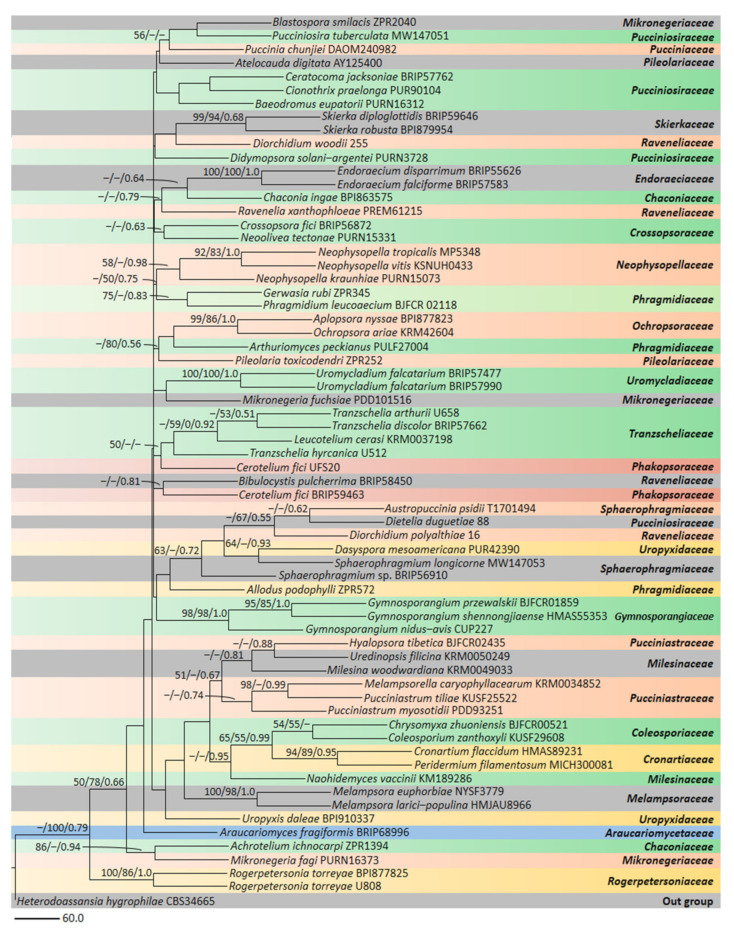
Phylogenetic placement of the rust genus *Skierka*, based on three genes ITS, LSU and SSU sequence data. The tree is rooted with *Heterodoassansia hygrophilae* (CBS34665) as the outgroup. The phylogenetic tree showed that the *Skierka diploglottidis* BRIP59646 and *Skierka robusta* BPI87995 (*Skierkaceae*) branch uniquely in the RAXML tree and branch with *Diorchidium woodii* 255 (*Raveneliaceae*) showing good bootstrap support at 99/94 in ML/MP/BI, respectively.

*Skierka* has long held an isolated placement within *Pucciniales*. Arthur [15,16] placed *Skierka* in a separate subfamily or tribe, respectively, in the *Pucciniaceae;* while Cummins and Hiratsuka [1] treated it as *incertae sedis* within the rusts. Mains [20] hypothesized that *Skierka* represented an intermediate taxon between *Melampsoraceae* and *Pucciniaceae* (equivalent to the subordinate ranks *Melampsorineae* and *Raveneliineae*/*Uredinineae*, under the present classification). However, with the incorporation of DNA-based molecular studies in addition to basic morpho-taxonomy, this genus has now gained a definite placement in the order *Pucciniales*. A general outline of rust fungi is presented here (Table 3 and Figure 2) to understand the accurate taxonomic position of the rust genus *Skierka*.

The order *Pucciniales* consists of 26 families and 147 genera, of which 9 families are incomplete and one (*Uncolaceae*) lacks complete molecular data. Among the 147 genera, 54 have no molecular data. We aim to drown at least two sequences from each family, first confirming whether the type genus has a sequence, if not we have chosen an alphabetical order of the genus. Most species have an LSU sequence, and the second most common is ITS; finally, the least common are SSU and *COX3*. In phylogeny, three gene regions, such as LSU (45), ITS (35) and SSU (25) are used instead of LSU, SSU, and the *COX3* combination [6] to create the multigene phylogenetic tree.

The ML tree based on three concatenated loci was mostly consistent with previous studies on more limited taxa and locus. The ITS, LSU, and SSU sequences were chosen to construct the multigene phylogeny. The DNA sequence data of various rust fungi including the *Skierka* species from the LSU, SSU and ITS rDNA were downloaded from GenBank and via previously published literature. Nucleotide sequences from ITS, LSU, and SSU were unambiguously aligned using the MAFFT v7.450 online server (https://mafft.cbrc.jp/alignment/server/, accessed on 4 October 2022), exported as aligned sequence data [37], and then manually checked and possibly edited in BioEdit v.7.0.9 [38]. The sequences of taxa containing poorly aligned parts, incomplete data, missing sequence data, and gaps have been removed. The separate aligned gene regions of ITS, LSU, and SSU were combined in BioEdit. The combined multigene sequence alignment was converted to the PHYLIP (.phy) format for a randomized accelerated maximum likelihood (RAxML) analysis. Matched ITS, LSU, and SSU single gene datasets and a concatenated dataset of LSU, ITS and SSU genes were converted to maximum likelihood using RAxML-HPC2 on XSEDE (8.2.8) [39] in the CIPRES Science Gateway platform [40] using the GTR+I+G evolution model. Maximum likelihood bootstrap values greater than 50% have been reported over each node. The phylogenetic trees were visualized using the program FigTree v1.4.0 [41] and reorganized in Microsoft PowerPoint. A checklist of molecular studies on various rust fungi, including *Skierka* spp., along with the name of the isolate, was also prepared and presented in Table 3.

MP was born with PAUP v. 4.0b10 [42] with the following parameters, such as unordered balanced characters, random addition of taxa, and branch swapping with a bisection Tree Reconnection Algorithm (TBR), which reduces branching when the maximum branch length is zero. Alignment gaps were treated as missing characters in the combined dataset analysis, where they occurred in relatively sheltered regions. The trees were derived using the heuristic search option with 1000 additions of random sequences, with the maximum number of trees fixed at 1000 descriptive tree Statistics for thrift. The length of the trees (TL), the consistency index (CI), the retention index (RI), the Relative Consistency Index (RC), and the Homoplasy Index (HI) were calculated for the trees, generated according to various optimization criteria. Kishino–Hasegawa tests [43] were performed to determine if the trees differed significantly.

The MB analysis, using MrBayes v.3.1.2 [44], was carried out for the assessment of the subsequent Bayesian probabilities (BYPP) [45], by sampling from Markov Chain Monte Carlo (BCMMC), and a GTR+I+G fit model was used in the command. Six simultaneous Markov chains were run over 1,000,000 generations and tree samples were taken every 1000 generations. The first 20% of the trees produced were discarded and the remaining 80% were used to calculate the subsequent probabilities of the majority rule consensus tree. A BYPP equal to or greater than 0.50 is reported on the nodes. We consider Bootstrap support equal to or higher than 75 as strong support, between 50 to 75 as moderate support, and below 50 is considered to be minimum support.

Multigene phylogeny of *Pucciniales* constructed by LSU, ITS and LSU regions of 45, 35 and 25 sequences, respectively. In the RAxML analysis, a minimum scoring tree was obtained with a final ML optimization probability value of −24539.275524. The array had 1370 distinct alignment patterns with 50.12% indeterminate characters or gaps. The estimated base frequencies were as follows: A = 0.292354, C = 0.168457, G = 0.248170, T = 0.291019, substitution rate AC = 1.279072, AG = 3.096291, AT = 1.979488, CG = 0.491674, CT = 4.918627, GT = 1.000000 invariant sites I = 0.233373, gamma distribution shape parameter α = 0.364863. The maximum parsimony data set consists of 2669 characters, of which 1396 were constants, 793 informative parsimony, and 480 non-informative parsimony. Parsimony analysis of the data matrix resulted in a thousand equally parsimonious trees with a tree length of 5047 steps (CI = 0.420, RI = 0.397, RC = 0.167, HI = 0.580) in the first tree. The general topology of the resulting ML phylogenetic tree is similar and consistent with previous studies [6]. The phylogenetic tree showed that *Skierka diploglottidis* BRIP59646 and *S. robusta* BPI87995 (*Skierkaceae*) branch uniquely in the RAXML tree and branch with *Diorchidium woodii* 255 (*Raveneliaceae*) showing good bootstrap support 99/94 in ML/MP/BI, respectively.

## 4. Taxonomy

**Figure 3:**
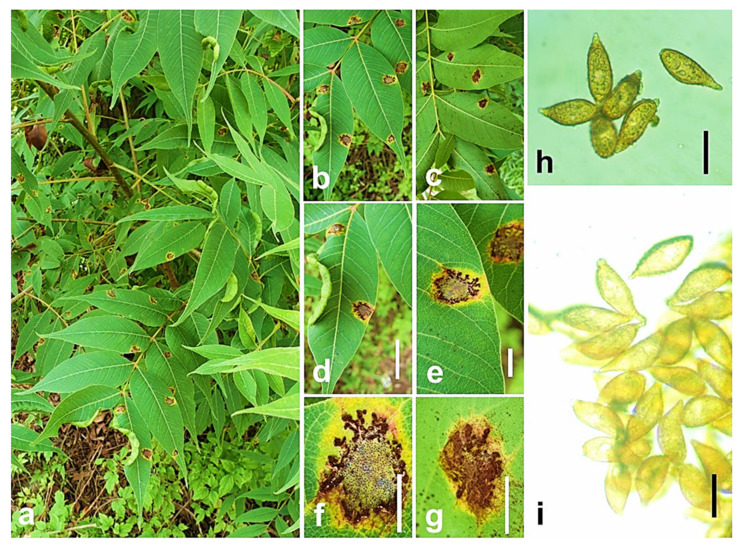
*Skierka himalayensis*. (**a**–**g**) Leaves of *Pistacia integerrima* showing rust infection (telia) on abaxial and adaxial surfaces; (**h**,**i**) Teliospores seen in LM. Scale bars: (**d**–**g**) = 1 mm; (**h**,**i**) = 10 μm.

**Figure 4:**
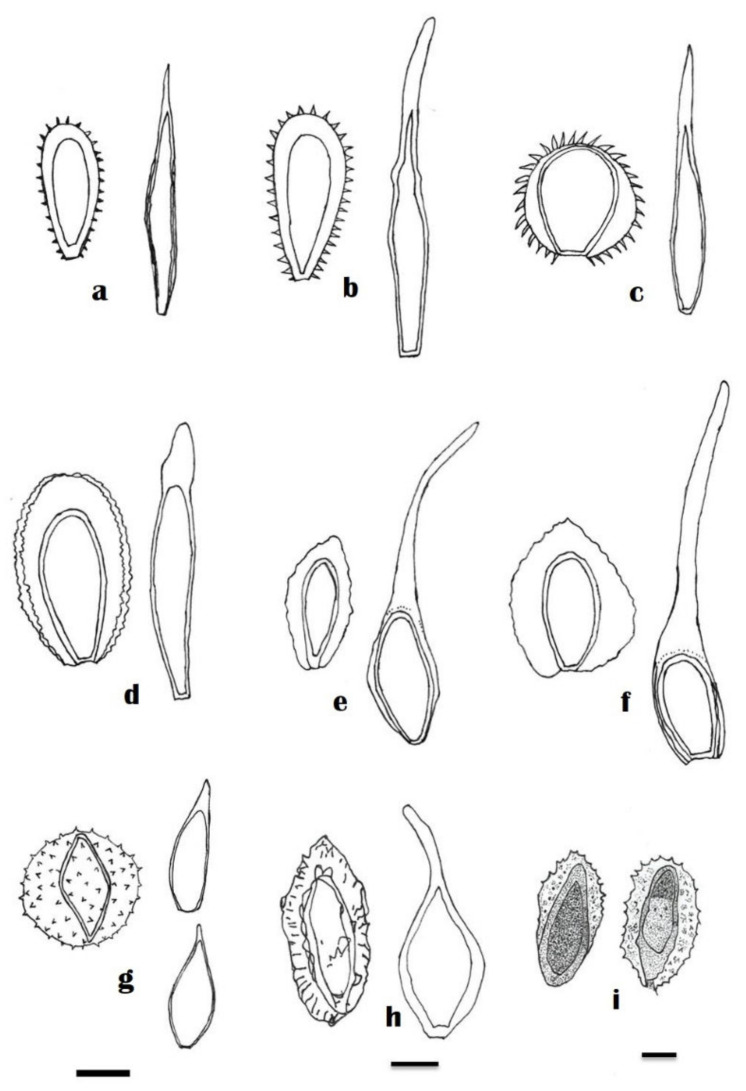
Uredospores and teliospores (line diagrams) different *Skierka* spp. (**a**) *S. congensis* Henn; (**b**) *S. philippinensis* Mains; (**c**) *S. canarii* Racib; (**d**) *S. cristata* Mains; (**e**) *S. petchii* (Syd.) Mains; (**f**) *S. holwayi* Arthur; (**g**) *S. nephelii* S. Ito and Muray; (**h**) *S. divinopolensis* Dianese; (**i**) *S. agallochae* Racib. Redrawn references [20,25,28,31,34]. Scale bars = 20 μm.

The genus *Skierka* was described by Raciborski [8] with the description of one-celled, fusoid teliospores of *Skierka cauarii* having acuminate apices, without pedicels, as a type species. There has been a long-term placement of this genus in families such as *Pucciniaceae*, *Melampsoraceae*, and *Pileolariaceae*, the subfamily *Skierkatae* of the *Aecidiaceae*, tribe *Skierkeae* of the *Pucciniaceae*, and the tribe *Hemileieae*. In the recent high-rank taxonomic framework proposed by Aime and McTaggart [6], this genus is finally placed in a separate family *Skierkaceae,* of the suborder *Skierkineae,* in the order *Pucciniales*. To understand the morphological and microscopical characteristics of each *Skierka* spp., the individual and comparative taxonomic description of these rust fungi based on the information available in published literature are presented in Figure 3 and Figure 4 and Table 4. 

Phylum: *Basidiomycota* R.T. Moore Bot. Mar. 23: 371 (1980)

Subphylum: *Pucciniomycotina* R. Bauer, Begerow, J.P. Samp., M. Weiss and Oberw., Mycol. Prog. 5: 45 (2006)

Class: *Pucciniomycetes* R. Bauer, Begerow, J.P. Samp., M. Weiss and Oberw., Mycol. Prog. 5: 48 (2006)

Order: *Pucciniales* Caruel, Atti R. Accad. Naz. Lincei, Mem. Cl. Sci. Fis. Matem. Nat., sér. 5: 246 (1881)

Suborder: *Skierkineae* Aime and McTaggart, Fungal Systematics and Evolution 7: 31 (2020)

Family: *Skierkaceae* (Arthur) Aime and McTaggart, Fungal Systematics and Evolution 7: 31 (2020)

Basionym: *Skierkatae* Arthur, North American Flora 7: 704 (1926) 

Type genus: *Skierka* Racib., Parasit. Alg. Pilze Javas (Jakarta) 2: 30 (1900)

Type Species: *Skierka canarii* Racib., Parasit. Alg. Pilze Java’s (Jakarta) 2: 30 (1900)

The new rust family *Skierkaceae* was proposed by Aime and McTaggart [6]. The family produces deep-seated and subepidermal sporothalli sori, with mature uredinio- and teliospores that are single-celled and non-catenulate. These spores are forced through an arrow sorus opening by the production of new spores from sporogenous cells, from which they are detached before extrusion. This distinguishes these rust fungi from all other rust fungi [6].

The general identifying characteristics of the genus *Skierka* includes *Uredinia* single-celled, subepidermal with a deep-seated opening by a pore, urediniospores with the wall thickened into two opposed longitudinal ridges or bands, the thickenings frequently expanding greatly in water. *Pycnia* subepidermal (occasionally deep-seated in hypertrophied regions). *Telia* is subepidermal, deep-seated, opening by a pore, *Teliospores* are fusoid, single-celled, have a colorless wall, and frequently have two layers, with the younger teliospores forming between the older ones and adhering to them. The spore mass typically pushes out of the telium as a thread or column. The rust disease symptoms are generally observed on mature leaves [6,20,30,46]. 

***Skierka agallochae*** Racib., Bull. int. Acad. Sci. Lett. Cracovie, Cl. Sci. Math. Nat. Sér. B, Sci. Nat. 3: 275 (1909) (Figure 4i)

= *Skierka agallocha* Racib. (1909)

The rust sori formed by the fungus are amphigenous showing small, yellow or pale brownish, scattered, loosely aggregated or arranged in a circle, round, minute (0.1–0.4 mm diam.), diffused, subepidermal deep-seated uredosporiferous sori, long covered with epidermis. *Uredospores* yellow-brown, brown or dark-brown, ovate, oblong, spindle-shaped or clavate (47.0–90.0 × 20.0–43.0 μm), covered with short thick spines loosely and evenly subhyaline or a pale yellow membrane showing obscure germ-pores. The teliospores have emerged through the opening of the telia in threads (50–80 μm wide and 1–8 mm long). *Teliospores* smooth, thin-walled, 8.0–12.0 × 60.0–100.0 μm in size with an 18–25 μm apex. *Pycnia* subepidermal epiphyllous, with ostiolar filaments and resembling type-5. Aecia subepidermal, deep-seated, hypophyllous, possibly peridiate associated with pycnia showing opening by a pore. *Aeciospores* golden yellow, echinulate, double layered wall, with two lateral ridges, borne singly on pedicels and 8.2–4.1 μm laterally, 41–69.7 × 20.5–32.8 μm, mostly 61.5 × 20.5–28.7 μm [20,23,34,35].

Hosts and distribution: On *Excoecaria agallocha* (*Euphorbiaceae*) var. *genuina* from Batavia, Java (Indonesia); Okinawa Islands (Japan); Maharashtra (India) in Asia.

Note: This rust fungus (*S. agallochae*) was first described by Raciborsky (1909) in the telial stage on *Excoecaria agallocha*. Later on, Mains (1939) [20] monographed this genus *Skierka* and provided the telial description of this fungal species. Chavan [34] described the first Indian record of this genus under *S. agallocha* and provided pycnial, aecial, and uredial stages, described for the first time for this rust species.

***Skierka canarii*** Racib., Parasit. Alg. Pilze Java’s (Jakarta) 2: 30 (1900) (Figure 4c).

The rust forms subepidermal, powdery rust pustules under the surface of the leaves (hypophyllous) as minute, subepidermal, covered by the epidermis except for a small pore, pulverulent uredinial sori scattered or in small groups; urediniospores obovoid, 14.0–16.0 × 24.0–30.0 μm, bounded by hyaline or yellowish, uniform in thickness (1.0–1.5 μm), the inner wall and the outer wall are 1.0–1.5 μm thick, thickening into two opposing longitudinal wings 2.5–6.0 μm broad, the edges provided with a fringe of curving teeth-like projections; the remaining portion of the spore is coarsely echinulate in longitudinal lines, with the wings in the optical plane having a circular shape and measuring 24–38 × 24–30 μm. *Telia* tiny, hypophyllous, dispersed or in small clusters, subepidermal, and covered by the overlying epidermis. *Teliospores* adhere and emerge as a short column from a small pore. They are fusoid, smooth-walled, hyaline, 11–19 × 64 –110 μm, then 1 μm or less acuminate apex, solid, 14–40 μm truncate base [8,20,35].

Hosts and distribution: On *Canarium commune*, *Canarium moluccanum*, *Canarium* sp. (*Burseraceae*) from Java (Indonesia), New Guinea in Oceania and on *Canarium villostttn* (the Philippines) in Asia.

Note: The species *Skierka canarii* was described as the type species of this genus by Raciborski (1900) [8]. Thereafter, it was reported by Koorders [19] from Java. The specimens reported and analyzed by Arther and Cummins [47] support the previous species identity. The rusts studied by Sydow and Sydow [48], Sydow and Petrak [49], and Sydow and Petrak [50], provided the records on *S. canarii* on *Canarium villostttn* for the Philippines. The presence of the fringe of thin, curved teeth on the borders of urediniospores, distinguishes this species from *S. philippinensis*. Teliospores emerge out through a small opening of the telium as a column, the younger spores are pushed up between the older ones but do not catenulate.

***Skierka clemensiae*** Cummins, Mycologia 33: 145 (1941)

The rust fungus forms amphigenous uredia and aggregate in spots 0.5–2.0 mm in diam, open pores 0.1–0.25 mm in diam. *Urediospores* ellipsoid, obovoid, more or less fusoid 10–15 (–17) × (20–)23–30(–33) μm in size with hyaline, moderately echinulate membrane, 1.5 μm having dark germ pores dark. *Telia* and *Teliospores* are still unknown [24].

Hosts and distribution: On *Canarium* sp. (*Burseraceae*) from New Guinea in Oceania.

Note: *Skierka clemensiae* was firstly described by George Baker Cummins in 1941, where he proposed it as a new species. As it was found to resemble *S. philippinensis*, Cummins [24] differentiated it by having smaller urediospores with walls of uniform thickness. The echinulation is more uniformly distributed with no marked tendency toward longitudinal arrangement.

***Skierka congensis*** Henn., Ann. Mus. Congo Belge, Bot., Sér. 52: 90 (1907) (Figure 4a)

The rust fungus forms scattered, sub-epidermal, subrounded powdery rust pustules, which later on developed into delicate white threads of spores forced from small pores of the infected part (leaves) of the host plant. *Uredia* developed as scattered gregarious yellow, pulverulent, subrounded spots scattered on the lower side of leaves (hypophyllous) or as groups of tiny, rounded, pale russets. *Uredospores* clavate or fusoid, echinato-verrucose, subhyaline and 23–38 × 8–16 μm in size bounded by 1.5 μm thick wall (up to 5.0 μm at the apex and 4.0 μ in two lateral longitudinal ridges) with obscure pores. *Telia* hypophyllous, subepidermal, in small groups, covered by the epidermis except for a small slit or pore. *Teliospores* lanceolate to fusoid, acute on both sides, hyaline, 50.0–85.0 × 6.0–8.0 μm, adhering and forced out in very long, delicate threads, thick and loosely coalesced [9,35].

Hosts and distribution: On the leaves of *Alchornea cordifolia*; *Macaranga* sp. (*Euphorbiaceae*) and *Dombeya* sp. (*Sterculiaceae*) from the Democratic Republic of the Congo and Sierra Leone in Africa.

Note: *Skierka congensis* was described by Paul Christoph Hennings in 1907. The infection of this fungus is only reported on hosts of two families *Euphorbiaceae* and *Sterculiaceae,* from two African countries, which might reflect their restricted distribution. This species has also been documented by Shaw [36] in Papua New Guinea.

***Skierka cristata*** Mains, Mycologia 31: 182 (1939) (Figure 4d)

= *Ctenoderma cristatum* (Speg.) Syd. and P. Syd. [as ‘cristata’], in Saccardo and Trotter, Syll. Fung. (Abellini) 23: 663 (1925)

= *Uredo cristata* Speg., Anal. Soc. Cient. Argent. 17: 119 (1884)

= *Uromyces cupaniae* Arthur and J.R. Johnst., Mem. Torrey Bot. Club 17: 131 (1918)

The fungi appeared on the infected host surface as rust pustules, deep-seated in hypertrophies of leaves with powdery, yellowish spores and later by delicate long white threads. The rust fungi form amphigenous pycnia, which are subepidermal oblate-spheroid, 100–200 μm broad, 80–100 μm thick, and include ostiolar filaments. They are clustered in hypertrophied patches that are 2–5 mm across. *Uredinia* are mostly hypophyllous, deeply embedded in hypertrophied tissue, covered by a thin layer of compacted hyphae beneath the epidermis, opening by a tiny hole, and pulverulent. *Urediniospores* are obovoid or fusoid, 16–20 × 30–40 μm bounded with a yellow and consistent in thickness inner wall (1.5–2.5 μm), the outer wall hyaline, swelling to form a longitudinal plate. The wings reaching 10–15 μm in width over the upper portion of the spore. *Spores* are obovate or fusiform in outline, 22–30 × 40–55 μm, coarsely and sparsely echinulate in the upper portion, crenate or serrate in lines on the edges of the lateral wings, the pores obscure, and the apices rounded or acute. *Telia* hypophyllous, dispersed or in tiny groupings, the spores adhere and are frequently pushed out in long, delicate white threads, similar to uredia. *Teliospores* are fusoid, 10–15 × 60–96 μm, and two-layered. The outer layer ultimately splits from the inner, with the apex acuminate and the base truncate [20,35].

Hosts and distribution: On *Cupania americana*, *Cupania belizonsis*, *Cupania macrophylla*, and *Cupania* sp. (*Sapindaceae*) from Cuba, Paraguay, Trinidad in South America, and Belize in North America.

Note: This rust was described by Mains [20] as the type species of the genus *Skierka*. Earlier, this species was identified as *Uromyces cupaniae* based on the observation that the urediniospores of this rust were teliospores. Similarly, Sydow and Sydow [51] also identified urediniospores of this species as teliospores and proposed this fungus as a separate genus *Ctenoderma* with this as the type species. However, the teliospores were re-assessed and confirmed as uredia again, and the number of teliospores was also found in these two species. Similarly, to the case of other *Skierka* species, the urediniospores of this species were thickened laterally with a thick plate surrounding the spore longitudinally except for the hilum. The crenate or serrate edges of the spores give them a cristate appearance, which perhaps justifies the name of this species [20].

***Skierka diploglottidis*** (Cooke and Massee) Mains, Mycologia 31: 184 (1939)

= *Coeomurus diploglottidis* (Cooke and Massee) Kuntze [as ‘Caeomurus’], Revis. Gen. Pl. (Leipzig) 3: 450 (1898)

= *Ctenoderma diploglottidis* (Cooke and Massee) Syd., Annls. Mycol. 20: 55 (1922)

= *Uromyces diploglottidis* Cooke and Massee, in Cooke, Grevillea 17: 55 (1889)

The rust fungus epiphyllous, subepidermal uredinia that are entirely covered by the epidermis and a dense layer of compacted hyphae (10–20 μm), except for a tiny hole or slit. *Urediniospores* oblong-fusoid, 12–16 × 32–42 μm, bounded by yellowish, 1.5–3.0 μm thick inner wall and hyaline outer wall, which thickened to form two opposite longitudinal lateral plates, with the plates in the optical plane. *Spores* are elliptic-fusiform in outline, 22–28 × 40–60 μm, the apices acute, and the edges of the plates crenate. Similarly, to uredia, the telia are subepidermal, covered by the epidermis and a dense layer of compacted hyphae except at the point of its opening. *Teliospores* are fusoid, 15.0–18 × 70.0–90.0 μm, with the acute apex and covered with a colorless, 1.5 μm thick wall [20].

Hosts and distribution: On *Dictyoneura obtuse* and *Diploglottis cunninghamii* (*Sapindaceae*) from Queensland in Australia, and Bailey (Texas) in North America.

Note: In the case of *S. cristata*, the teliospores of this fungus are also identified as the urediospores of *Uromyces* [52], *Coeomurus* [53], and *Ctenoderma* [54]. The detailed study of [20] solves this confusing placement of this fungi and placed it under the genus *Skierka*. In addition, the molecular characterization of this rust fungus reported on *Dictyoneura obtusa* based on three gene regions (SSU, LSU, and *COX3*) along with one more species of *Skierka* was carried out by Aime and McTaggart [6]. This study resolved the long-going confusion of the taxonomic placement of this genus. As a result, this species was placed under a new suborder *Skierkineae* and a new rust family *Skierkaceae*.

***Skierka divinopolensis*** Dianese, R.B. Medeiros and L.T.P. Santos, Fitopatol. Brasil. 18: 446 (1993) (Figure 4h)

= *Uromyces diploglottidis* Cooke and Massee, in Cooke 1889

The fungus produced a few round, crowded, hypophyllous, light brown uredia (216–260 µm in diam.) sunken in mesophyll with a light surrounding peridium, the upper part of erumpent, and form short columns of light yellow to whitish spore mass. *Urediniospores* produced are ellipsoidal to fusoid with a truncate base [(50–) 55–77 × (24–) 28–32 (–34) µm in size] covered 1.0 mm thick wall, except for a truncate base, thickening laterally to 6.0–8.0 µm to form a band around the urediniospores. The band surface form moderate crenate or minute echinulation. *Germ pore* obscure. *Telia* produced hypophyllous, subepidermal, and erumpent in overgrown leaf tissue and usually in small groups. *Teliospores* adhering to each other to form columns 1.4–4.8 × 48–60 µm, *Teliospores* fusoid (35–) 43–48 (–50) × (15–) 17–20 µm with hyaline to pale yellow 1–2 µm thick smooth wall; apex acuminate and base truncate, germinal apical without resting period forming an external cylindrical metabasidium (48–52 × 7–8 µm in size). *Spermogonia* and *Acacia* are unknown [28].

Hosts and distribution: On leaves of *Cupania rugosa*, *Matayba guianensis* (*Sapindaceae*) from Minas Gerais (Brazil) in South America.

Note: The specific epithet “*divinopolensis*” was named after the city Divinopolis, located in the state of Aminas Gerais where the first specimen of the fungus was collected. As described by Dianese et al. [28], this species is differentiated from *S. cristata* [20] in terms of morphology and the size of the teliospores and basidiospores.

***Skierka himalayensis*** A.K. Gautam and S. Avasthi, Acta Mycologica 52: 2 (2017) (Figure 3).

The fungus appeared as rust pustules on the undersurface of leaves (hypophyllous), initiated as small brown to blackish, rounded rust sori, surrounded by a reddish yellow or chlorotic zone. The pustules initially scattered and later coalesced to form a hard dry crust on the leaf surface. *Telia* subepidermal; teliospores one-celled, sessile, 35.7–48.3 (mean ± SD, 41.26 ± 3.99) × 10.5–18.9 (mean ± SD, 14.28 ± 3.10) μm in size, walls 2.5–3.5 μm thick. *Teliospores* produced in irregular succession, strongly adherent and extruded in long, hair-like columns [30].

Host and distribution: On mature leaves of *Pistacia integerrima* (*Anacardiaceae*) from Mandi, Himachal Pradesh (India) in Asia.

Notes: The new species as *Skierka himalayensis* was proposed after a comparison of morphologically similar species, namely *S. canarii* Racib. and *S. petchii* (Syd.) Mains. The major variation was observed in the dimensions of the teliospores. The teliospores of *S. himalayensis* showed variation in their size, wall thickness, and size of the beak in comparison with the other two studied species [30].

***Skierka holwayi*** Arthur, Am. J. Bot. 5: 433 (1918) (Figure 4f)

This rust fungus forms yellowish rust pustules with loose spore horns initially on the lower leaf surface and later in long columns on both surfaces of the leaves (amphigenous). *Pycnia*, sub-epidermal, discoid, 350–450 μm wide, 90–130 μm thick, and formed in small groups. Primary uredia developed as in groups surrounding the pycnia, mostly epiphyllous, flask-shaped, developing immediately beneath the greatly enlarged epidermal cells, covered by a layer of compacted hyphae beneath the epidermis and opening by a pore, secondary uredia rare, scattered, sub-epidermal but the epidermal cells are not enlarged. *Urediniospores* occasionally develop in spore horns, which easily crumble in water, narrowly obovoid (14–20 × 30–65 μm), bounded by a yellowish brown, uniformly thickened (2.0–2.5 μm) inner wall. The outer wall is hyaline, swelling laterally to form a plate reaching 26–36 μm in width longitudinally, surrounding the spore except for the hilum. *Spores* with this plate in the optical plane appeared ovate or cordate in outline, 26–36 × 30–65 μm, acute at the apex, and smooth except for the edges of the plate, which are irregularly crenate. *Telia* is mostly hypophyllous, developed with pycnia and primary uredinia, developed in groups (sometimes scattered), and then does not cause enlargement of the epidermal cells. *Teliospores* adhering, fusoid, 11–14 × 28–38 μm forming long yellowish columns. Exclusive of the very slender apex, teliospores reaching a length of 60 μm have smooth, hyaline, two wall layers, the inner 1.5 μm, the outer 1 μm or less, separating from the inner [20].

Host and distribution: On leaves of *Thouinidium decandrum* and *Thouinidium* sp. (*Sapindaceae*) from Guatemala in North America.

Note: The fungus was first described in 1918 [11] on leaves of *Thouinidium* spp. This rust contains many well-developed characteristics, which are either undeveloped or not well-developed in many other species of this genus, and was considered the most unusual rust.

***Skierka nephelii*** S. Ito and Muray., Trans. Sapporo Nat. Hist. Soc. 17: 165 (1943) Figure 4g)

= *Uredo nephelii* (S. Ito and Muray.) Hirats. f., Trans. Mycol. Soc. Japan 2: 11 (1959)

This fungus forms rust pustules on the lower surface of the leaves of Litchi chinensis. Initially, uredia appeared on the lower leaves surface (hypophyllous). Similar to uredia, telia is also observed on the lower leaves surface, scattered or irregularly aggregated, covered with epidermis for a long time up to the eruption of short, filamentous, and white teliospores. *Teliospores* adhering to each other but easily retiring, unicellular, ellipsoid, obovoid, elongate-obovoid, elongate pyriform or subfusiform, 23−75 × 12−20 µm, base usually truncate, apex obtuse or pointed beak sharp or forming obtuse, hyaline, leaves, walls lateral ca. 1 µm or less thick, usually at the tip, thickened up to 15 µm thick, pores germination indistinct (Ito and Murayama 1943; Zhuang et al. 2021) [25,31].

Host and distribution: On *Litchi chinensis* (*Sapindaceae*) from Taiwan, China in Asia.

Note: The rust was first described by Ito and Murayama in 1943 [25] based on taxonomic characteristics of uredia and urediospores. In the study carried out by Zhuang et al. [31], both uredia (urediospores) and telia (teliospores) were observed and their detailed taxonomic examination was performed.

***Skierka petchii*** (Syd.) Mains, Mycologia 31: 185 (1939) (Figure 4e)

= *Ctenoderma petchii* Syd., Annls. Mycol. 21: 342 (1923)

This fungus appeared as reddish-brown rust sori on the leaves of *Sapindus* spp. At the beginning of the rust disease, uredia appeared on both surfaces of the leaves (amphigenous) as reddish-brown spots, which are sub-epidermal and covered by the epidermis except for a small pore or slit. *Urediniospores* narrowly ellipsoid (8−12 × 24−50 μm) as bounded by a yellowish (1.5−2.0 μm thick) inner wall, the hyaline outer wall, which thickened to form two lateral irregularly crenate edged longitudinal wings. *Urediniospores* fusiform in outline, 18−20 × 39−60 μm, the apices acute. Further, telia developed similarly to uredia, which produce obovoid-fusoid, excluding the apex, 12−18 × 38−44 μm. *Teliospores,* which adhere to form short columns, the apex is long attenuate, 25.0−50.0 μm long, the wall hyaline, the inner 1.0 μm, the outer thinner and separating from the inner wall [20].

Host and distribution: *Sapindus bifoliolatus* (*Sapindaceae*) from India and Sri Lanka, and in Asia.

Note: This rust fungus was identified as *Uredo cristata* Speg., while Sydow (1923) [55] described it as a species of *Ctenoderma*. In this description, urediniospores were described as teliospores. Further, when a few teliospores were found to be associated, uredia were studied in detail and observed to be closely related to the genus *Skierka*. This fungus was also found to be closely related to *S. holwayi,* based on the characteristics of the urediniospores. However, the lateral thickenings of the wall of the urediniospores and the long attenuate apices of the teliospores led to the identity of the former species.

***Skierka philippinensis*** Mains, Mycologia 31: 180 (1939) (Figure 4b)

The rust forms subepidermal, pulverulent rust pustules, later with a loose column on the leaves of *Canarium* spp. Initially, the formation of scattered, hypophyllous, subepidermal, pulverulent uredia (0.2−0.5 mm across) was observed on leaves, covered by the epidermis and a thin layer of compacted hyphae except for a small pore or slit. *Uredinospores* ellipsoid or ellipsoid-fusoid, 12−16 × 28−54 μm in size, covered with a moderately echinulate wall layer with variable thickening. The wall layer is generally 1.0−1.5 μm thick, 4−6 μm laterally in a band extending longitudinally around the spore except for the hilum, pores obscure. *Telia* formed scattered (0.2–0.5 mm across) on lower leaves surface (hypophyllous), subepidermal, adhering and forced out in irregular loose columns; *Teliospores* fusoid with long acuminate apex, truncate base (11−19 × 64−110 μm) bounded with a wall, smooth, hyaline (1.5−2.5 μm thick), the outer layer often separating from the inner [20].

Host and distribution: On *Canarium luzonicum* and *Canarium* sp. (*Burseraceae*) from the Philippines in Asia, and Papua New Guinea in Oceania.

Note: This rust was described by Mains [20] on *Canarium* sp. from the Philippines and Papua New Guinea. Initially, it was identified as *Skierka canarii*. However, characteristics of the urediniospores, teliospores, edged lateral wings, and echinulations on the wall resembled *S. philippinensis* more, hence, it was renamed as this species of *Skierka*.

***Skierka robusta*** Doidge, Bothalia 2: 155 (1927)

Telia observed on *Rhoicissus rhomboidea* from South Africa. *Teliospores* are given as yellowish, narrow-lanceolate or lanceolate-fusiform, 20–27 × 120–180 μm, acuminate, elongated into a long filiform process, covered with a 3.0–3.5(–5.0) μm thick wall. *Uredia* are unknown [12].

Host and distribution: On *Rhoicissus rhomboidea* (*Vitaceae*) from South Africa.

Note: This fungus was identified and described by Doidge [12] on *Rhoicissus rhomboidea* based on the morpho-taxonomy of the rust spores. Recently, a molecular characterization based on two gene regions (SSU and LSU) was carried out by Aime and McTaggart [6], which provided an updated taxonomic position of this species under a new suborder *Skierkineae* and a new rust family *Skierkaceae*.

***Skierka toddaliae*** (Petch) Hirats., in Ito and Murayama, Trans. Sapporo Nat. Hist. Soc. 17: 165 (1943)

= *Aecidium toddaliae* Petch, Ann. R. Bot. Gard. (Peradeniya) 4: 303 (1909)

= *Ctenoderma toddaliae* (Petch) Syd. and P. Syd., Annls. Mycol. 17: 103 (1919–1920)

= *Didymopsora toddaliae* Thirum. and Mundk., Proc. Indian Acad. Sci., Sect. B 16: 170 (1942)

= *Didymopsorella toddaliae* (Petch) Thirum., Sci. Cult. 16: 210 (1950)

= *Uredo toddaliae* Petch, in Sydow, Fungi exotici exsiccati, Fascicle 2: 69 (1913)

The rust fungus appeared as amphigenous, discoid pycnia grouped in yellowish spots (150−200 μm wide, 50−75 μm thick) without filaments. Later on, uredinia surround the pycnia and form hypophyllous uredesori, deep-seated in the host tissue, having a very angular opening via a small pore or slit. *Uredeniospores* (28−36 × 40−70 μm) surrounded by double wall layers. The inner wall is uniform (1.5 μm thick), and yellowish-brown whereas, the outer hyaline wall forms a very thin layer on most of the spore. The irregular thickening of the outer wall (up to 10 μm thick) forms ridges of various extents mostly over the apex or at the base (occasionally from the base to apex). The outer wall showed irregular and fine echinulation, especially on the ridges. *Telia* stage is still unknown [25].

Host and distribution: On *Toddalia aculeate* (*Rutaceae*) from Sri Lanka in Asia.

Note: This species was described by Ito and Murayama [25] based on the morpho-taxonomy of pycnia and uredinia. The absence of the telial stage and irregular thickening of the outer spore wall in ridges pointed out that the placement of this rust lay under the genus *Skierka.* Therefore, further studies based on DNA techniques are required to resolve the taxonomic placement.

## 5. Conclusions

Beginning with the first report of *Skierka canarii* as the type species of the genus *Skierka* by Raciborski [8], a total of 14 species have been described so far; however, only *Skierka himalayensis* has been reported in the 21st century. Further analyses revealed that the maximum number of species were reported during 1900–1910 (3), 1931–1940 (4), and 1941–1950 (3). Single species was reported during the second (1911–1920) and third decades (1921–1930), respectively; thereafter, the next report was observed after 43 years, i.e., 1993. After that, a new species *Skierka* was reported again after a huge gap of 24 years, i.e., in the year 2017. Likewise, the morpho-taxonomy of all species has been well studied, but molecular analyses are still required. Only two species of the genus namely *S. robusta* and *S. diploglottidis* have been identified at the molecular level, the multiple gene analysis was carried out by Aime and McTaggart [6] based on 18S small subunit ribosomal rRNA (SSU), 28S large subunit ribosomal rRNA (LSU), and Cytochrome C oxidase III (*COX3*) gene regions. The importance of molecular studies for all species of the genus *Skierka* can be predicted from the study by Aime and McTaggart [6], where they proposed a new and separate family to accommodate the genus in *Skieraceae* instead of *Pileolariceae*. Due to the lack of molecular studies, many genera or species need to be recollected and epitypified, to place them in their correct taxonomic position. The combination of traditional and modern methods is an important approach and is a demand of our time to understand fungi (rust fungi) more precisely [56,57]. As the molecular phylogeny is carried out, the taxonomic ambiguities of all these species will be resolved.

As the plant family, *Sapindaceae* (total of eight hosts) was found to be infected with *Skierka* sp., the specificity of these fungi towards these hosts can be explored further at the genomics and metabolomics level. The occurrence of these on six plant families over 19 countries across the globe provides an outlook on their vast distribution; however, with most of the reports coming from Asian regions, compared with no report from any European region, this may reflect their adaptability towards geographical regions and climatic conditions. However, the European regions should be explored more for the presence of fungi including rust fungi (*Skierka* sp.). Therefore, further studies should be focused on epitypifying many species of herbarium samples as well as carrying out fresh collections of these rust fungi based on molecular, biochemical, and physiological aspects along with their morphological characteristics. Importance should also be given to unveiling the relationship between rust pathogens and host preference in order to understand this fungal genus more precisely. These findings will enhance the understanding of the identification, diversity, and distribution of these rust fungi.

## Figures and Tables

**Table 1:** The detailed history of the genus *Skierka*.

Year	Research Detail	Reference
1900	The genus *Skierka* was proposed based on the species *Skierka cannarii*	[8]
1907	*Skierka congensis* was described as the second species of this genus	[9]
1907	*Skierka* was placed in the *Melampsoraceae* family	[19]
1907	A subfamily *Skierkatae* of the *Aecidiaceae* (*Pucciniaceae*) was proposed and the genus *Skierka*, along with *Ctenoderma*, *Sphenospora*, and *Chaconia*, were included	[15]
1909	*Skierka agallocha* was described as a new species from Java	[10]
1915	*Skierka* was placed in *Pucciniaceae* based on the fusoid teliospores that resemble *Uromyces*-like rust	[13]
1918	*Skierka holwayi* was described as a new species from Central America	[11]
1925	*Skierka* was included as the oldest group of the *Pucciniaceae*	[22]
1926	*Skierka robusta* was described as a new species from Africa	[12]
1928	*Skierka* was placed in the tribe *Skierkeae* of the *Pucciniaceae*	[16]
1939	*Skierka cristata* was described. This species was derived from the re-examination of the rust sample of *Ctenoderma cristata* (Speg.) Sydow (*Uredo cristata* Speg.)	[20]
1939	*Skierka diploglottidis* was described as a new species	[20]
1939	*Skierka petchii* was described as a new species	[20]
1939	*Skierka philippinensis* was described as a new species	[20]
1940	*Skierka agallocha* was described from the Ryukyu Islands	[23]
1941	*Skierka clemensiae* was described as a new species	[24]
1943	*Skierka nephelii* was described as a new species	[25]
1943	*Skierka toddaliae* was described as a new species	[25]
1954	*Skierka agallocha* was reported from the Ryukyu Islands	[26]
1981	The host range *Skierka* was described with *Sapindaceae*, *Rutaceae*, *Burseraceae*, and *Euphorbiaceae*	[27]
1993	*Skierka divinopolensis* was described as a new species	[28]
1998	SEM studies of *Skierka agallocha* on mangrove rust of Sundarbans, Eastern India	[29]
2017	*Skierka himalayensis* was described as a new species	[30]
2021	Telial stage of *Skierka nephelii* was reported as a new rust addition to subtropical China	[31]
2022	Reviewed the contributions to Cerrado mycology (including *Skierka*) from the early 19th century to date	[32]

**Table 2:** List of described *Skierka* species, together with the host (family), and country of distribution.

Name	Host (Family)	Biogeographical Region (Country)	References
*Skierka agallochae* Racib.	*Excoecaria agallocha* (*Euphorbiaceae*)	Batavia, Java (Indonesia), Okinawa Islands (Japan), and Maharashtra (India)	[10,23,26,33,34]
*Skierka canarii* Racib.	*Canarium commune*, *Canarium moluccanum*, *Canarium villostttn*, and *Canarium* sp. (*Burseraceae*)	Java (Indonesia), New Guinea, and the Philippines (Asia)	[8]
*Skierka clemensiae* Cummins	*Canarium* sp. (*Burseraceae*)	Papua New Guinea	[24]
*Skierka congensis* Henn.	*Dombeya* sp. (*Sterculiaceae*), *Alchornea cordifolia*, and *Macaranga* sp. (*Euphorbiaceae*)	The Democratic Republic of the Congo, and Sierra Leone	[9,20]
*Skierka cristata* Mains	*Cupania americana*, *Cupania belizonsis*, *Cupania macrophylla*, and *Cupania* sp. (*Sapindaceae*)	Cuba, Paraguay, Trinidad, and Belize	[20]
*Skierka diploglottidis* (Cooke and Massee) Mains	*Dictyoneura obtuse* and *Diploglottis* sp.(*Sapindaceae*)	Queensland and Bailey (Texas)	[20]
*Skierka divinopolensis* Dianese	*Cupania rugosa* and *Matayba guianensis* (*Sapindaceae*)	Minas Gerais (Brazil)	[28]
*Skierka himalayensis* A.K. Gautam and S. Avasthi	*Pistacia integerrima* (*Pistaceaceae*)	Himachal Pradesh (India)	[30]
*Skierka holwayi* Arthur	*Thouinidium decandrum*, and *Thouinidium* sp. (*Sapindaceae*)	Guatemala	[11]
*Skierka nephelii* S. Ito and Muray.	*Litchi chinensis* (*Sapindaceae*)	Taiwan (China)	[25,31]
*Skierka petchii* (Syd.) Mains	*Sapindus bifoliolatus* (*Sapindaceae*)	Sri Lanka	[20]
*Skierka philippinensis* Mains	*Canarium luzonicum* and *Canarium* sp. (*Burseraceae*)	Philippines and Papua New Guinea	[20,35,36]
*Skierka robusta* Doidge	*Rhoicissus rhomboidea* (*Vitaceae*)	South Africa	[12]
*Skierka toddaliae* (Petch) Hirats.	*Toddalia aculeate* (*Rutaceae*)	Sri Lanka	[25]

**Table 3:** GenBank and voucher or culture collection accession numbers of rust fungal species were included in the phylogenetic study.

Taxon	Voucher Number (Collection Number)	GenBank Accession No.		
		LSU	ITS	SSU
*Achrotelium ichnocarpi*	ZPR1394	MK518684	MK518985	MK488400
*Allodus podophylli*	ZPR572	MK518482	MK518834	MK488140
*Aplopsora nyssae*	BPI877823	MW049244	NA	NA
*Araucariomyces fragiformis*	BRIP68996	MW049245	NA	MW049292
*Araucariomyces fragiformis*	BRIP68996	NG074475	NA	NG073560
*Arthuriomyces peckianus*	PULF27004	NA	MW448622	NA
*Atelocauda digitate*	-	NA	NA	AY125400
*Austropuccinia psidii*	T1701494	NA	MK020421	NA
*Baeodromus eupatorii*	PURN16312	MW049246	NA	NA
*Bibulocystis pulcherrima*	BRIP58450	MW049247	NA	NA
*Blastospora smilacis*	ZPR2040	MK518730	MK519028	MK488480
*Ceratocoma jacksoniae*	BRIP57762	KT199394	NA	KT199382
*Cerotelium fici*	BRIP59463	MH047210	NA	MW049299
*Cerotelium fici*	UFS20	NA	MZ047090	NA
*Chaconia ingae*	BPI863575	MW049249	NA	NA
*Chrysomyxa zhuoniensis*	BJFCR00521	MZ444061	NR153462	NA
*Cionothrix praelonga*	PUR90104	MW049252	NA	NA
*Coleosporium zanthoxyli*	KUSF29608	MH460677	MH465095	NA
*Cronartium flaccidum*	HMAS89231	MK208289	MK193822	NA
*Crossopsora fici*	BRIP56872	MH047208	NA	MH047213
*Dasyspora mesoamericana*	PUR42390	NA	NR136010	NG064973
*Didymopsora solani argentei*	PURN3728	MW049254	NA	NA
*Dietelia duguetiae*	88	NA	KM217365	KM217382
*Diorchidium woodii*	255	KM217352	NA	KM217370
*Gerwasia rubi*	ZPR345	MK518735	NA	MK488442
*Gymnosporangium przewalskii*	BJFCR01859	NG060667	NR154073	NA
*Heterodoassansia hygrophilae*	CBS34665	NG064047	NR160101	NA
*Hyalopsora tibetica*	BJFCR02435	NG081469	MK795976	NA
*Leucotelium cerasi*	KRM0037198	KX228776	KX228771	NA
*Melampsora euphorbiae*	NYSF3779	MK518509	MK518852	MK488184
*Melampsora larici populina*	HMJAU8966	MT757879	MT759611	NA
*Melampsorella caryophyllacearum*	KRM0034852	ON063363	ON063389	NA
*Mikronegeria fagi*	PURN16373	MW049267	NA	MW049298
*Mikronegeria fuchsiae*	PDD101516	NA	KX985772	NA
*Milesina woodwardiana*	KRM0049033	NA	NR163315	NA
*Naohidemyces vaccinii*	KM189286	NA	MZ159489	NA
*Neoolivea tectonae*	PURN15331	NG074476	NA	NG073561
*Neophysopella vitis*	KSNUH0433	OM420271	OM423812	NA
*Ochropsora ariae*	KRM42604	KX228778	KX228773	NA
*Peridermium filamentosum*	MICH300081	MK208299	MK193831	NA
*Phragmidium leucoaecium*	BJFCR02118	MN264737	MN264719	NA
*Pileolaria toxicodendri*	ZPR252	MK518537	MK518871	MK488231
*Pucciniastrum myosotidii*	PDD93251	NA	KJ716347	KJ746815
*Pucciniastrum tiliae*	KUSF25522	OL519197	OL519191	NA
*Puccinia chunjiei*	DAOM240982	NA	NR111548	NA
*Pucciniosira tuberculate*	-	MW147051	NA	NA
*Ravenelia xanthophloeae*	PREM61215	MG946017	MG945985	NA
*Rogerpetersonia torreyae*	BPI877825	NG075238	NA	NG073502
*Rogerpetersonia torreyae*	U808	MG907207	NA	NA
*Skierka diploglottidis*	BRIP59646	MW049278	NA	MW049304
*Skierka robusta*	BPI879954	MW049279	NA	MW049305
*Sphaerophragmium longicorne*	-	MW147053	NA	MW147077
*Sphaerophragmium* sp.	BRIP56910	KJ862350	NA	KJ862429
*Tranzschelia arthurii*	U658	MG948659	MG947386	NA
*Tranzschelia discolor*	BRIP57662	KR994891	NA	KR994969
*Tranzschelia hyrcanica*	U512	NA	MG948663	NA
*Uredinopsis filicina*	KRM0050249	MK302213	MH908488	NA
*Uromycladium falcatarium*	BRIP57477	KJ632973	NR138392	KJ633013
*Uropyxis daleae*	BPI910337	NA	KY798364	NA

**Table 4:** A comparative account of the morphological characteristics of *Skierka* species.

Species	Taxonomic Characteristics				Reference
	Uredia (mm)	Uredospores (µm)	Telia (mm)	Teliospores (µm)	
*Skierka agallochae* Racib.	0.1–0.4	20–43 × 47–90	0.1–0.8 × 0.5–0.8	8–12 × 60–100	[10]
*Skierka canarii* Racib.	-	14–16 × 24–30	-	7–10 × 65–75	[8]
*Skierka clemensiae* Cummins	0.5–2.0	10–15 × 23–30	-	-	[24]
*Skierka congensis* Henn.	-	8–16 × 23–38	-	6–8 × 50–85	[9]
*Skierka cristata* Mains	-	16–20 × 30–40	-	10–15 × 60–96	[20]
*Skierka diploglottidis* (Cooke and Massee) Mains	-	12–16 × 32–42	-	15–18 × 70–90	[20]
*Skierka divinopolensis* Dianese	21.6–26	(24–) 28–32 (–34) × (50–) 55–77	-	(15–) 17–20 × (35–) 43–48 (–50)	[28]
*Skierka himalayensis* A. K. Gautam and S. Avasthi	-	-	-	10.5–18.9 × 35.7–48.3	[30]
*Skierka holwayi* Arthur	-	14–20 × 30–65	-	11–14 × 28–38	[11]
*Skierka nephelii* S. Ito and Muray.	-	-	-	12−20 × 23−75	[25]
*Skierka petchii* (Syd.) Mains	-	8–12 × 24–50	-	12–18 × 38–44	[20]
*Skierka philippinensis* Mains	0.2–0.5	12–16 × 28–54	0.2–0.5	11–19 × 64–110	[20]
*Skierka robusta* Doidge	-	-	-	20–27 × 120–180	[12]
*Skierka toddaliae* (Petch) Hirats.	-	28–36 × 40–70	-	-	[25]

## Data Availability

Not applicable.
